# Pharmacophore Modeling and Virtual Screening for the Discovery of New type 4 cAMP Phosphodiesterase (PDE4) Inhibitors

**DOI:** 10.1371/journal.pone.0082360

**Published:** 2013-12-10

**Authors:** Miaomiao Niu, Fenggong Dong, Shi Tang, Guissi Fida, Jingyi Qin, Jiadan Qiu, Kangbo Liu, Weidong Gao, Yueqing Gu

**Affiliations:** Department of Biomedical Engineering, School of Life Science and Technology, China Pharmaceutical University, Nanjing, China; Aligarh Muslim University, India

## Abstract

Type 4 cAMP phosphodiesterase (PDE4) inhibitors show a broad spectrum of anti-inflammatory effects in almost all kinds of inflamed cells, by an increase in cAMP levels which is a pivotal second messenger responsible for various biological processes. These inhibitors are now considered as the potential drugs for treatment of chronic inflammatory diseases. However, some recently marketed inhibitors e.g., roflumilast, have shown adverse effects such as nausea and emesis, thus restricting its use. In order to identify novel PDE4 inhibitors with improved therapeutic indexes, a highly correlating (r = 0.963930) pharmacophore model (Hypo1) was established on the basis of known PDE4 inhibitors. Validated Hypo1 was used in database screening to identify chemical with required pharmacophoric features. These compounds are further screened by using the rule of five, ADMET and molecular docking. Finally, twelve hits which showed good results with respect to following properties such as estimated activity, calculated drug-like properties and scores were proposed as potential leads to inhibit the PDE4 activity. Therefore, this study will not only assist in the development of new potent hits for PDE4 inhibitors, but also give a better understanding of their interaction with PDE4. On a wider scope, this will be helpful for the rational design of novel potent enzyme inhibitors.

## Introduction

 Type 4 cAMP-specific phosphodiesterase (PDE4) are a family of low k_m_ 3',5'-cyclic adenosine monophosphate (cAMP)-specific phosphodiesterases containing more than 20 isozymes encoded by four genes (PDE4A, PDE4B, PDE4C, and PDE4D) in mammals [1]. Even though four subfamilies share the conserved catalytic domain, each PDE4 gene plays a very important role in controlling the cell functions. PDE4s are taken as critical regulators of intracellular cAMP levels, cAMP signaling, and signal compartmentalization by their wide tissue distribution as well as differential expression and regulation among various cell types [1]. Thus many PDE4 inhibitors have showed remarked anti-inflammatory potential, by increasing cAMP levels. Recently the use of some newly marketed PDE4 inhibitors such as roflumilast, have been restricted due to their nausea and emesis. Therefore, the major pharmaceutical research focus in the field of chronic inflammatory diseases treatments, is to develop novel PDE4 inhibitors with high therapeutic index [1,2]. In our study, we successfully used pharmacophore modeling, database screening, and molecular docking approaches in identifying lead candidates to be used in potent PDE4 inhibitor design and thereby devising a new class of safer and effective anti-inflammatory agents. 

## Results and Discussion

### Pharmacophore modeling

**Figure 1:**
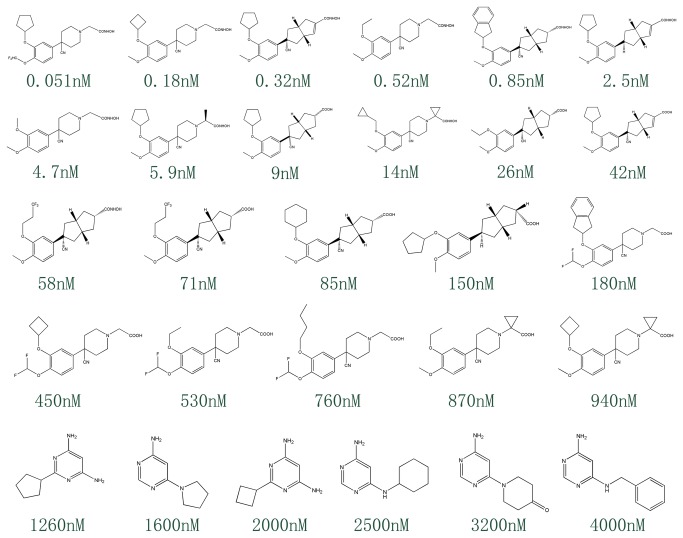
Chemical structures of PDE4 inhibitors in the training set.

**Figure 2:**
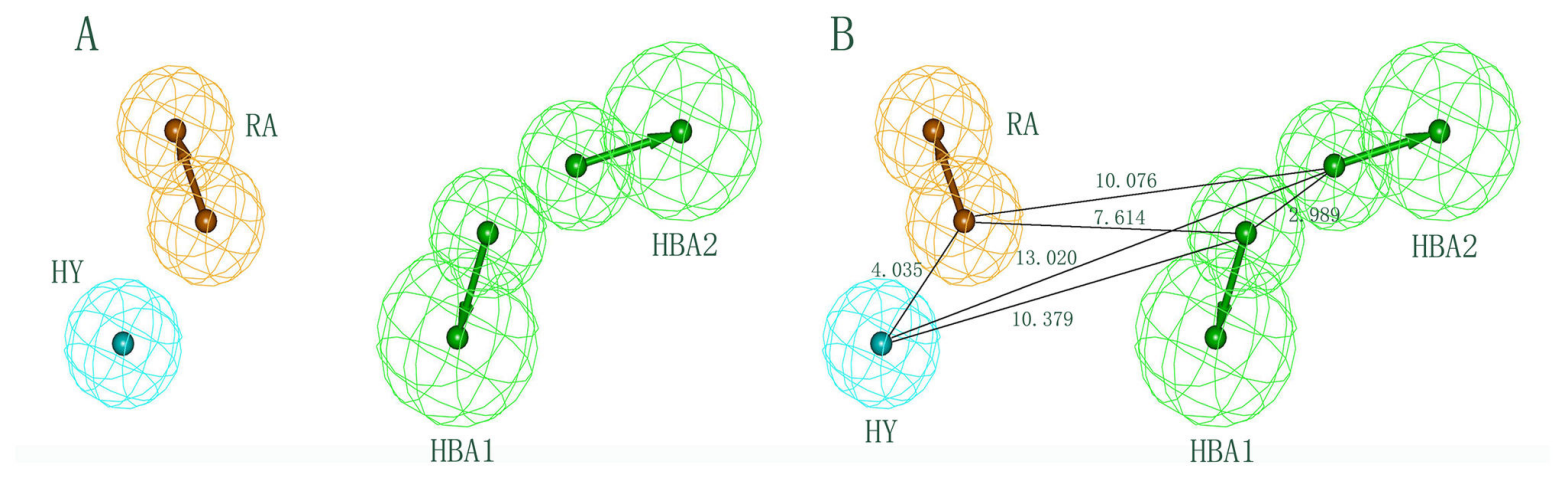
The best HypoGen pharmacophore model, Hypo1. (A) Chemical features present in Hypo 1. (B) 3D spatial relationship and geometric parameters of Hypo1. Pharmacophore features are color-coded (cyan, hydrophobic; orange, ring aromatic; green, hydrogen bond acceptor). Pharmacophore features are color-coded: cyan, hydrophobic (HY); orange, ring aromatic (RA); green, hydrogen bond acceptor (HBA).

**Figure 3:**
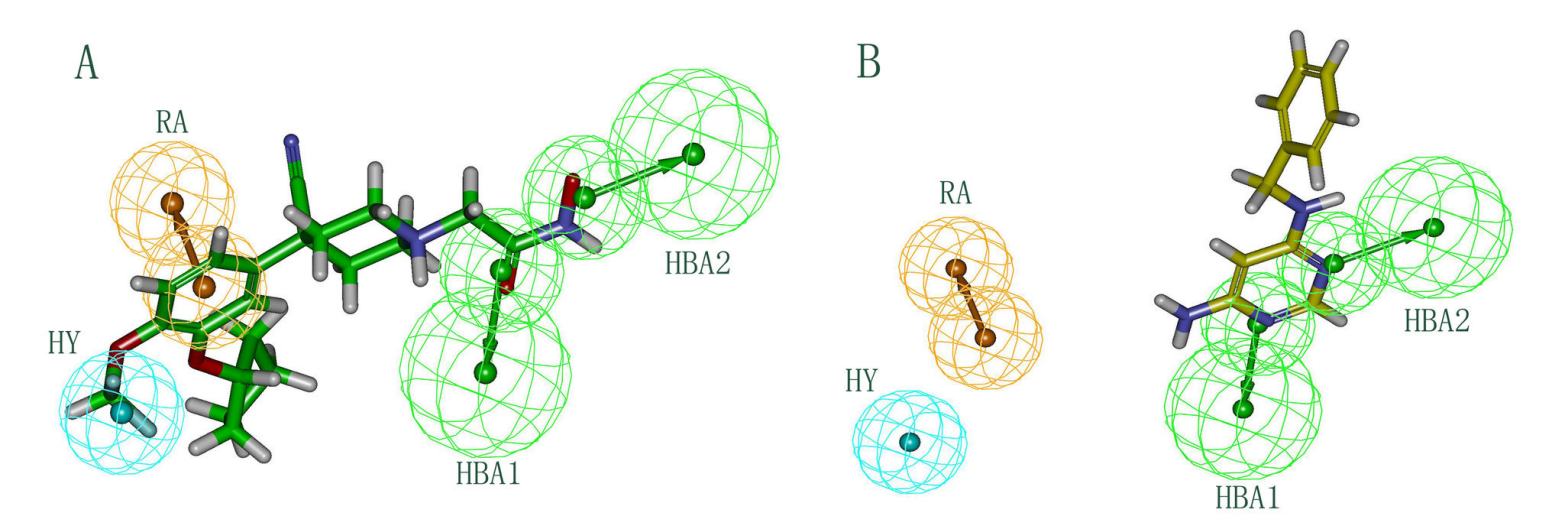
Pharmacophore Mapping. (A) Mapping of the most active compound 1 on the best pharmacophore model, Hypo1. (B) Mapping of the least active compound 28 on the best pharmacophore model, Hypo1. Pharmacophore features are color-coded: cyan, hydrophobic (HY); orange, ring aromatic (RA); green, hydrogen bond acceptor (HBA).

A set of ten pharmacophore models was generated by a training set containing 28 compounds. Structures of the training set compounds are shown in Figure 1. The total cost values of ten pharmacophore models ranged from 106.849 to 120.562 (Table 1). The cost difference between the null cost and total cost must be greater and it should be smaller between fixed cost and total cost values for a good pharmacophore model. In the present work, the first pharmacophore model (Hypo1) is basically composed of four features: two hydrogen bond acceptors, one hydrophobic region and one aromatic ring feature (Figure 2). Hypo1 was developed with a fixed cost value of 99.761 and a null cost value of 204.947. Among the total cost values of ten pharmacophore models, Hypo1 scored the closest value to the fixed cost value than other models. The cost difference for the first pharmacophore model was 98.098. A cost difference value above 60 implies that the pharmacophore model correlates the estimated and experimental activity values more than 90% [9,10]. Therefore, Hypo1 could be considered as a good model. Based on the correlation coefficient, ten pharmacophore models were further evaluated. The correlation values of the generated pharmacophore models were greater than 0.91, and the values for the first three pharmacophore models were even higher, i.e., above 0.950. These results implied the capability of the pharmacophore model to predict the activity of the training set compounds. Hypo1 showed the highest correlation coefficient value of 0.963930, indicating its strong predictive ability. Moreover, RMSD values for ten pharmacophore models were less than 1, further supporting the predictive ability of these models. Among the ten pharmacophore models, Hypo1 was developed with better statistical values, such as higher correlation, larger cost difference and lower RMSD. Based on the experimental activity (IC_50_) values, training set and test set compounds were categorized in following four groups: Highly active (IC_50_ < 10nM, ++++), active (10≤ IC_50_ < 200nM, +++), moderately active (200≤ IC_50_ < 1000 nM, ++), and inactive (IC_50_ ≥1000nM, +) [10]. Table 2 shows that activity values of all 28 compounds in the training set were predicted within their experimental activity scale, indicating the predictability of Hypo1. The pharmacophore mapping of most and least active compounds is shown in Figure 3. The most highly active compound (0.051 nM) mapped all the features of Hypo1, and the least active compound (4000 nM) missed hydrophobic and ring aromatic features. The reliability of Hypo1 has been further revealed. 

**Table 1:** Statistical results of the top 10 pharmacophore hypotheses generated by HypoGen algorithm.

Hypothesis	Total cost	Cost difference**^*a*^**	RMSD	Correlation	Features
Hypo1	106.849	98.098	0.53586	0.963930	HBA, HBA, HY, RA
Hypo2	110.479	94.468	0.58484	0.957944	HBA, HBA, HY
Hypo3	111.652	93.295	0.59607	0.952531	HBA, HBA, HBA
Hypo4	112.733	92.214	0.63861	0.949526	HBD, HY, HY
Hypo5	115.391	89.556	0.66088	0.946132	HBA, HBA, HBA
Hypo6	116.238	88.709	0.69439	0.938954	HBA, HBA, HBA
Hypo7	117.641	87.306	0.72386	0.931788	HBA, HBD, HY
Hypo8	118.967	85.980	0.78047	0.929203	HBA, HBD, HY
Hypo9	119.145	85.802	0.85013	0.924316	HBD, HY, HY
Hypo10	120.562	84.385	0.89086	0.917282	HBA, HBA, HY

Null cost = 204.947; fixed cost = 99.761; configuration cost = 15.383.

^a^ Cost difference = null cost – total cost; Abbreviations used for features: HBA, hydrogen-bond acceptor; HBD, hydrogen-bond donor; HY, hydrophobic region; RA, ring aromatic.

**Table 2:** Experimental and estimated IC_50_ values of the training set compounds based on best pharmacophore hypothesis Hypo1.

	IC_50_ nM			Activity scale**^*c*^**	
Name	Experimental**^*a*^**	Estimated	Error**^*b*^**	Experimental	Estimated
1	0.051	0.09	+1.8	++++	++++
2	0.18	0.34	+1.9	++++	++++
3	0.32	1.5	+4.7	++++	++++
4	0.52	0.43	-1.2	++++	++++
5	0.85	2.3	+2.7	++++	++++
6	2.5	1.9	-1.3	++++	++++
7	4.7	5.6	+1.2	++++	++++
8	5.9	3.8	-1.6	++++	++++
9	9	5	-1.8	++++	++++
10	14	10	-1.4	+++	+++
11	26	50	+1.9	+++	+++
12	42	62	+1.5	+++	+++
13	58	48	-1.2	+++	+++
14	71	98	+1.4	+++	+++
15	85	75	-1.1	+++	+++
16	150	130	-1.2	+++	+++
17	180	185	+1.0	+++	+++
18	450	380	-1.2	++	++
19	530	410	-1.3	++	++
20	760	850	+1.1	++	++
21	870	730	-1.2	++	++
22	940	620	-1.5	++	++
23	1260	1730	+1.4	+	+
24	1600	2300	+1.4	+	+
25	2000	2800	+1.4	+	+
26	2500	3300	+1.3	+	+
27	3200	2700	-1.2	+	+
28	4000	3600	-1.1	+	+

^a^References [3–8].

^b^Positive value indicates that the estimated IC_50_ is higher than the experimental IC_50_; negative value indicates that the estimated IC_50_ is lower than the experimental IC_50_.

***^c^***Activity scale: IC_50_ < 10nM (Most active, ++++); 10 ≤ IC_50_ < 200nM (Active, +++); 200 ≤ IC_50_ < 1000nM (Moderately active, ++); ≥ 1000nM (Inactive, +).

### Validation of the pharmacophore model

**Figure 4:**
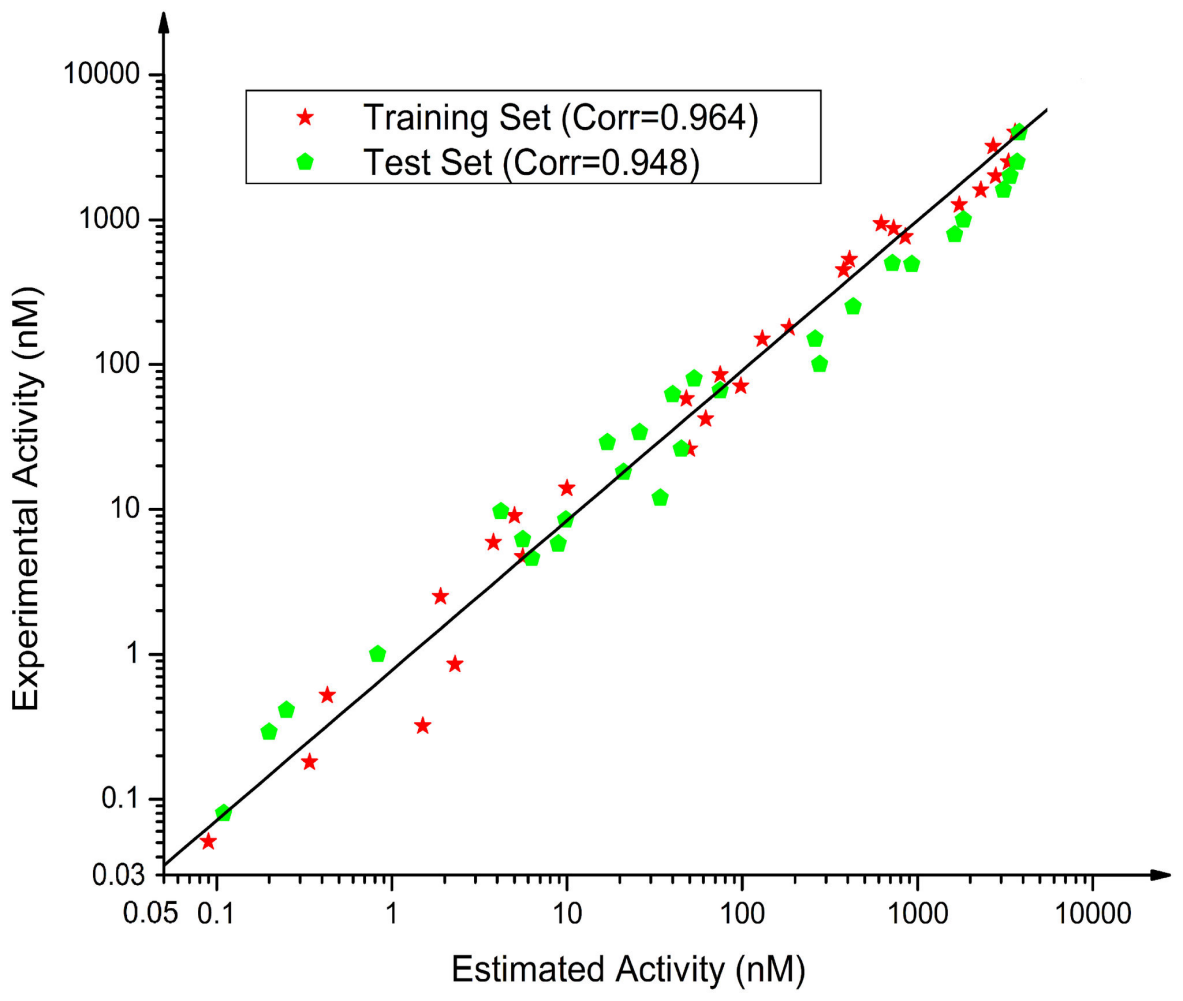
The correlation graph between experimental and estimated activity values based on Hypo1.

**Figure 5:**
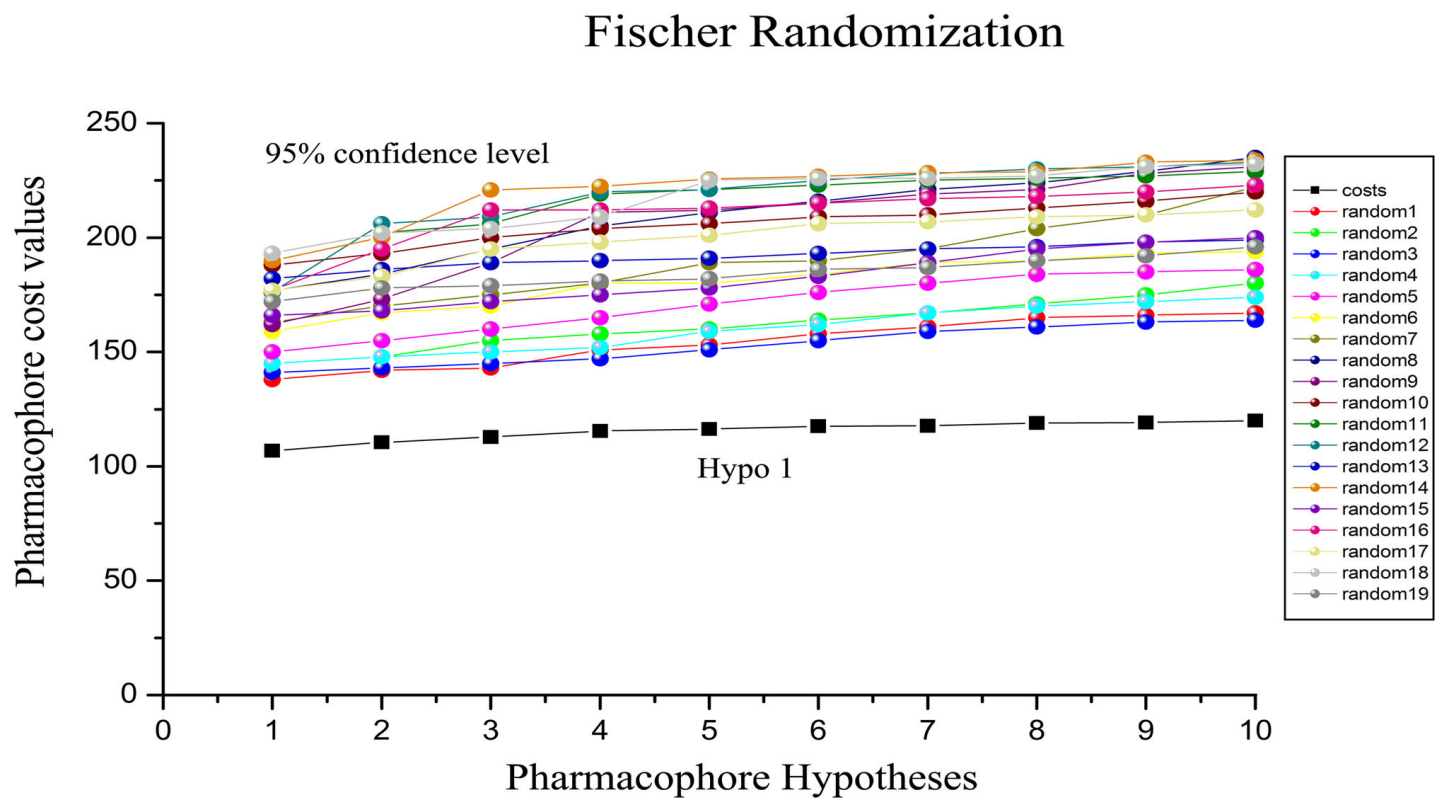
Results of Fischer randomization test for 95% confidence level.

The validation process was performed by a test set of 28 compounds with diverse activity classes and different functional groups. Diverse conformers of these test set compounds were built in the same manner as for training set compounds using DS. Based on the geometric fit of these compounds over Hypo1, the estimated activity values were predicted for every test set compound. A correlation coefficient value of 0.948 is shown by the simple regression between the experimental and estimated activity values of training and test set compounds (Figure 4). Two compounds out of 30 test set compounds were predicted in a different activity scale with a success rate of 93%. (Table 3). Especially, twenty-nine compounds from 30 test set compounds had error values less than 2.8, which was hardly different from the experimental and estimated activity values. The result showed a fairly good correlation between the experimental and estimated IC_50_ values, indicating a good predictive capacity of Hypo1. Fischer’s randomization method was additionally performed on the training set compounds to validate the statistical robustness of Hypo1. In this validation process, the experimental activities of the training set were scrambled randomly and the resulting training set was used in HypoGen module with the parameters chosen for the original pharmacophore generation [10]. To achieve a 95% confidence level, a set of 19 random spreadsheets is generated (Figure 5). The result clearly indicated that none of the randomly generated pharmacophore models obtained from this validation method was produced with better statistical values than Hypo1. The result of Fischer’s randomization test confirmed the statistical confidence of Hypo1. In order to verify whether the correlation between the experimental and estimated activities was mainly dependent on one particular compound in the training set, leave-one-out method was further used to perform the final validation. This was finished by recomputing the pharmacophore mode where one compound was excluded at a time. Under the same conditions which were used in the generation of the original pharmacophore model, 28 HypoGen calculations were carried out on 28 new training sets. The results indicated that compared to Hypo1, all the 28 new models generated by this method didn’t have any meaningful difference. This result confirmed the confident level of Hypo1 that its correlation coefficient didn't depend on one particular compound in the training set. 

**Table 3:** Test set compounds listed with their experimental, estimated activities and error values.

	IC_50_ nM			Activity scale**^*c*^**	
Name	Experimental**^*a*^**	Estimated	Error**^*b*^**	Experimental	Estimated
1	0.08	0.11	+1.4	++++	++++
2	0.29	0.2	-1.5	++++	++++
3	0.41	0.25	-1.6	++++	++++
4	1.0	0.83	-1.2	++++	++++
5	4.6	6.3	+1.4	++++	++++
6	5.8	8.9	+1.5	++++	++++
7	6.2	5.6	-1.1	++++	++++
8	8.5	9.8	+1.2	++++	++++
9	9.7	4.2	-2.3	++++	++++
10	12	34	+2.8	+++	+++
11	18	21	+1.2	+++	+++
12	26	45	+1.7	+++	+++
13	29	17	-1.7	+++	+++
14	34	26	-1.3	+++	+++
15	62	40	-1.6	+++	+++
16	66	75	+1.1	+++	+++
17	80	53	-1.5	+++	+++
18	100	180	+1.8	+++	+++
19	150	260	+1.7	+++	++
20	250	430	+1.7	++	++
21	490	930	+1.9	++	++
22	500	720	+1.4	++	++
23	790	1630	+2.1	++	+
24	1000	1832	+1.8	+	+
25	1600	3100	+1.9	+	+
26	2000	3385	+1.7	+	+
27	2500	3700	+1.5	+	+
28	4000	3800	+1.0	+	+

^a^References [3–8].

^b^Positive value indicates that the estimated IC_50_ is higher than the experimental IC_50_; negative value indicates that the estimated IC_50_ is lower than the experimental IC_50_.

***^c^***Activity scale: IC_50_ < 10nM (Most active, ++++); 10 ≤ IC_50_ < 200nM (Active, +++); 200 ≤ IC_50_ < 1000nM (Moderately active, ++); ≥ 1000nM (Inactive, +).

### Database screening and drug-likeness prediction

**Figure 6:**
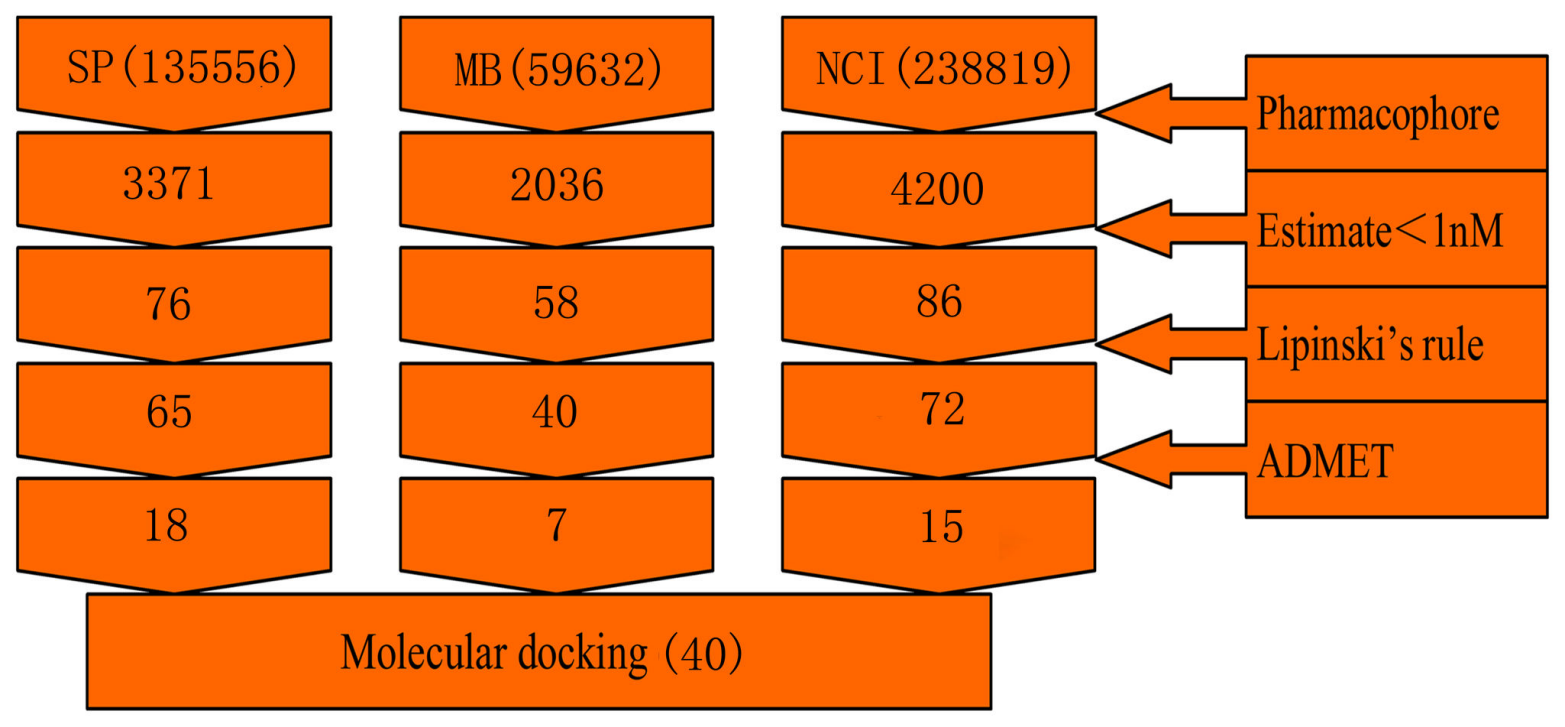
Database screening. The flowchart of procedure used in 3D QSAR pharmacophore modeling.

 Based on the above validation results, the selected Hypo1 was used as a 3D query to search chemical databases including Specs (135556 molecules), Maybridge (59652 molecules) and NCI (238819 molecules), containing totally 434027 compounds. The inhibitory activity values of these compounds were estimated. A total of 220 compounds were firstly screened by restricting the minimum estimated activity to 1 nM. Then on the basis of Lipinski’s rule of five and ADMET properties, these compounds were further screened to a number of 40. Finally, we subjected these 40 drug-like compounds along with the training set compounds to molecular docking study. Figure 6 lists the steps of the database screening procedure.

### Docking study

**Figure 7:**
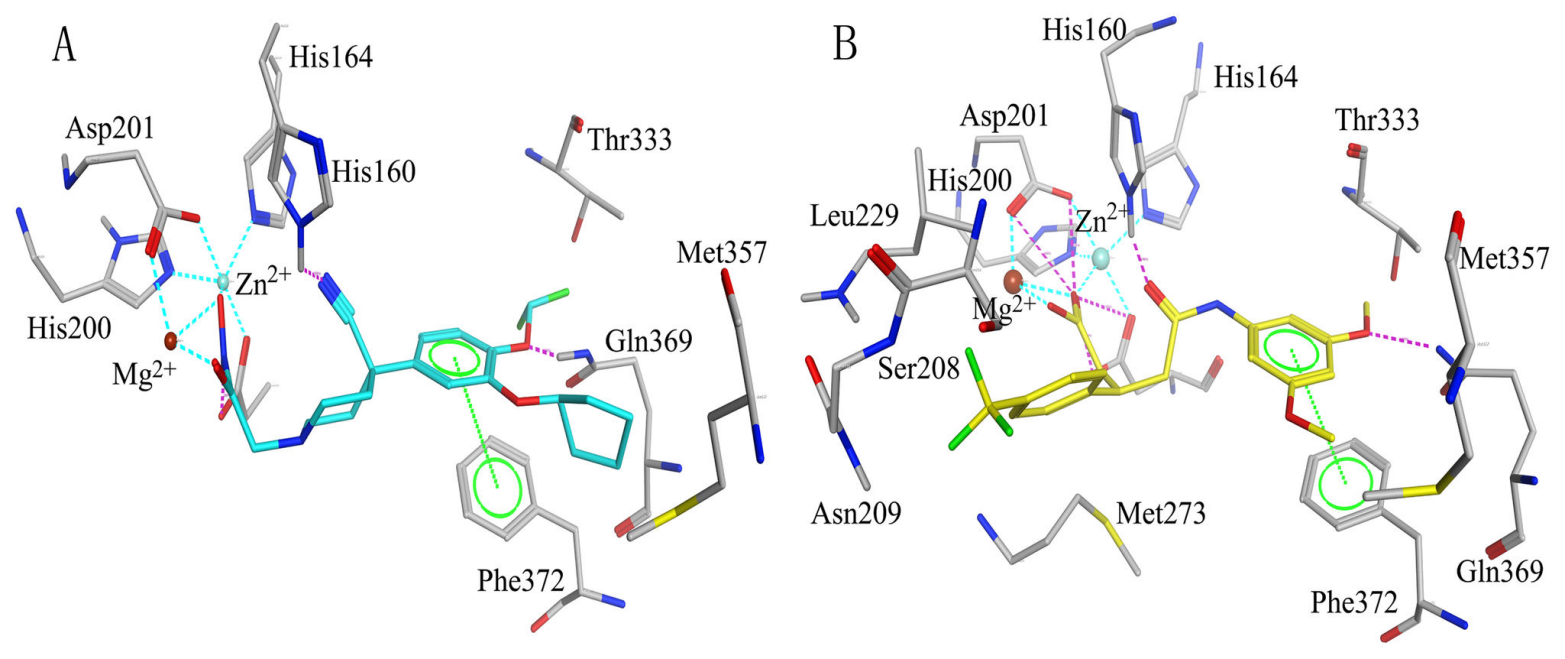
Molecular docking experiments of PD00519 and compound 1 in the training set. (A) Interaction between PDE4 and compound 1 as predicted by molecular docking. (B) Interaction between PDE4 and PD00519 as predicted by molecular docking. Active site residues are shown in stick form. Zn^2+^ and Mg^2+^ ions are shown in cyan and saddlebrown sphere, respectively. Hydrogen bond network with protein residues is represented in red dotted lines. Interaction network with metal ions is represented in cyan dotted lines. π-π stacking interaction is represented in green dotted lines. Compound 1 and PD00519 are color-coded: cyan – compound 1, yellow – PD00519.

**Figure 8:**
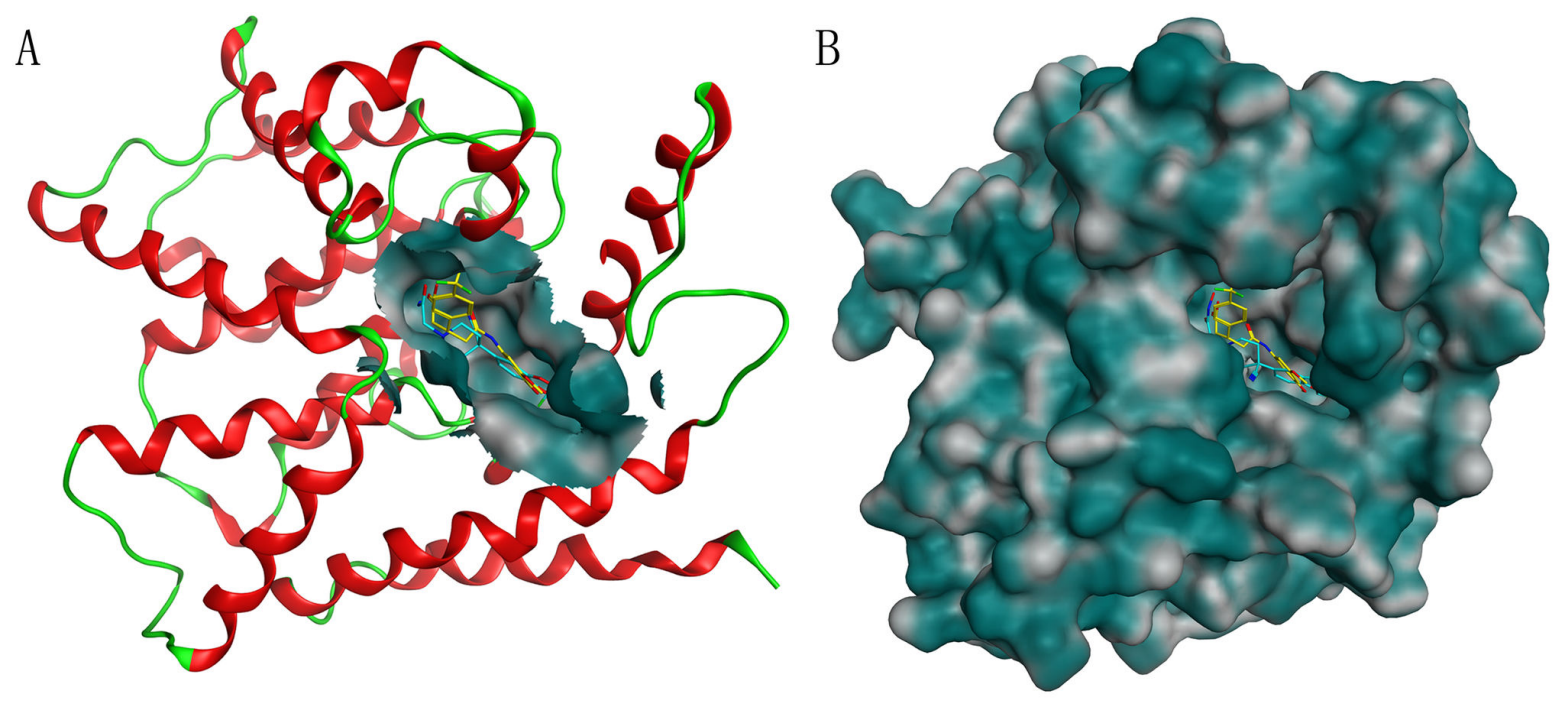
The positions of PD00519 and compound 1 as predicted by molecular docking. (A) Molecular overlay of PD00519 and compound 1 shown at the active site of PDE4. (B) The docking positions of PD00519 and compound 1 in the crystal structure of PDE4. PD00519 and compound 1 are color-coded: yellow – PD00519, cyan – compound 1.

**Figure 9:**
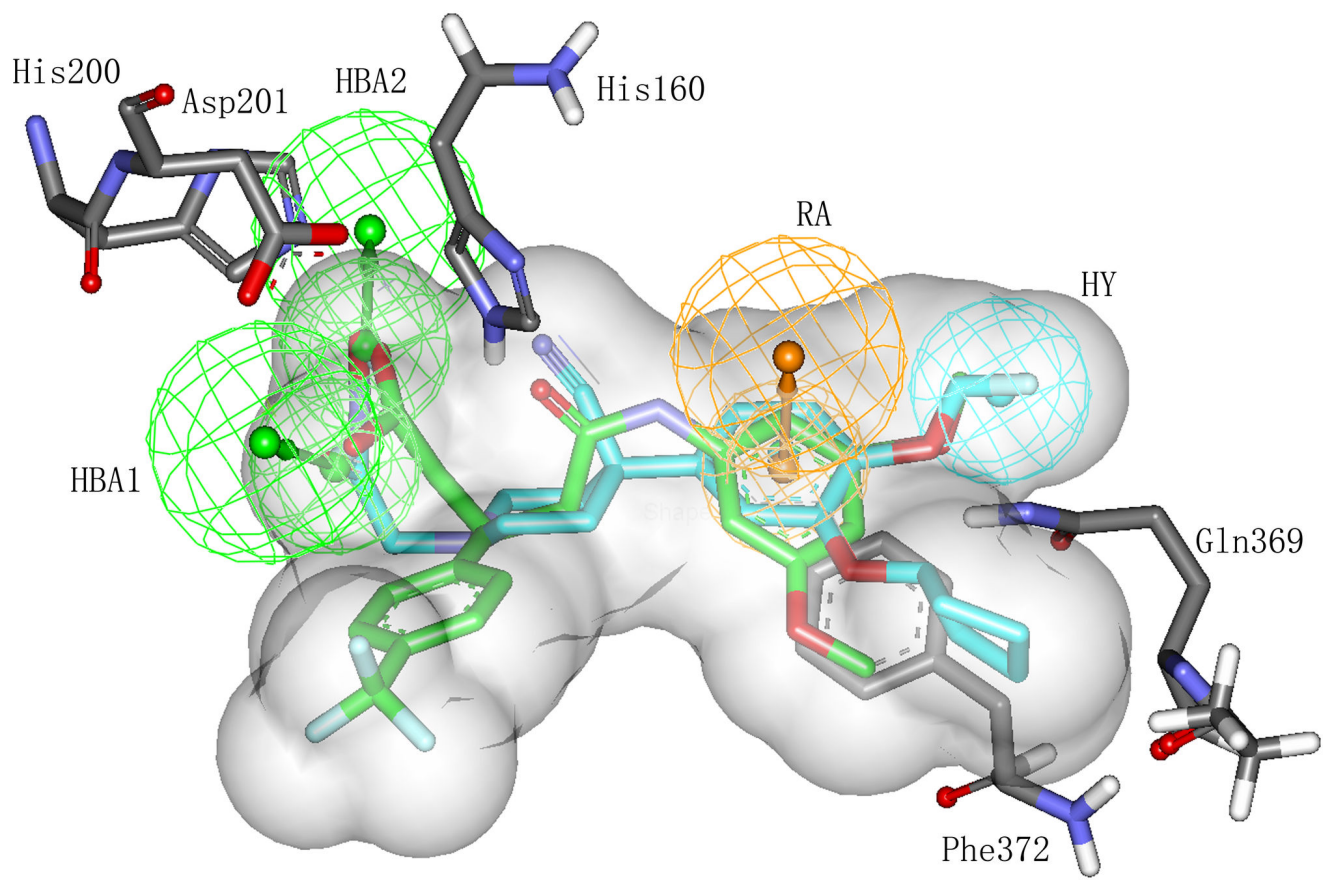
Pharmacophore mapping of PD00519 and compound 1 as predicted by molecular docking. Active site residues are shown in stick form. PD00519 and compound 1 are color-coded: green – PD00519, cyan – compound 1. Pharmacophore features are color-coded: cyan, hydrophobic (HY); orange, ring aromatic (RA); green, hydrogen bond acceptor (HBA).

 To further refine the retrieved hits, forty drug-like hit compounds along with the training set compounds were docked into the active site of PDE4. The active site was defined based on the bound inhibitor in a crystal structure of PDE4 (PDB entry: 1XON). The docking poses were ranked by the binding free energy calculation. The binding free energy and molecular interactions with the active site residues were considered as important components in selecting the best hit compounds. The most active compound of training set (compound 1) with the binding free energy of -8.897 kcal/mol, has formed hydrogen bond interactions with Gln369 and His160 and ionic interactions with Zn^2+^ and Mg^2+^ (Figure 7A). Moreover, this compound also exhibited a very important π-π interaction with the benzene ring of Phe372 and hydrophobic interactions with Met357, Thr333 and Gln369. On the basis of the molecular interaction of compound 1 and PDE4, we selected twelve drug-like compounds with a binding free energy lower than -8.897 kcal/mol of compound 1 as final hits for further evaluation process. Intriguingly, these hits were obtained from Maybridge and Specs databases. Table 4 shows the list of twelve hits along with estimated activity values. The estimated activity value of every hit was less than 0.2 nM and the binding free energy was lower than -9.0 kcal/mol. Particularly, the first hit, PD00519, obtained from Maybridge database, had an estimated activity value of 0.091 and the lowest free energy of -11.671 kcal/mol. The molecular interaction between PDE4 and PD00519 is shown in Figure 7B. The carboxyl group of PD00519 formed ionic interactions with metal ions (Mg^2+^ and Zn^2+^) while the dimethyl group formed hydrophobic interactions with Met357, Thr333 and Gln369. PD00519 had also formed a vital π−π interaction with Phe372 and hydrogen bond interactions with Gln369 and His160 in the active site of PDE4. The molecular docking study indicated that compared with compound 1 in training set, the benzotrifluoride of PD00519 that was fitted adequately into the hydrophobic pocket of PDE4 has formed hydrophobic interactions with Met273, Leu229 and Ser208 in the active site. The understanding of this interaction between PDE4 and PD00519 will be beneficial to develop new design for novel PDE4 inhibitors. Figure 8A shows overlay of compound 1 and PD00519 in the active site of PDE4 while Figure 8B indicates their docking positions in the crystal structure of PDE4. Figure 9 represents a fairly good pharmacophore mapping of PD00519 and compound 1 on Hypo1 as predicted by molecular docking. The oxygen atoms of PD00519 and compound 1 that formed important interactions with metal ions in the pocket overlaid two HBA features of Hypo1. Moreover, the benzene rings of PD00519 and compound 1 having π−π interactions with Phe372 mapped RA features of Hypo1, while the hydrophobic groups mapped the HY features of Hypo1. 

**Table 4:** List of twelve hit compounds from databases and their estimated activity values with the binding free energy.

Name	Estimated Activity (nM)	The binding free energy**^*a*^**
PD00519	0.091	-11.671
GK03776	0.131	-11.062
BTB01176	0.137	-10.745
AW01131	0.142	-10.114
AA-768-32245030	0.148	-10.031
AA-504-32628026	0.152	-9.878
AA-516-12432156	0.158	-9.742
BTB01888	0.166	-9.461
BTB01889	0.167	-9.125
BTB01180	0.179	-9.110
AW00861	0.183	-9.098
AA-768-30891048	0.190	-9.012

^a^ The docking poses were ranked by the binding free energy calculation.

**Figure 10:**
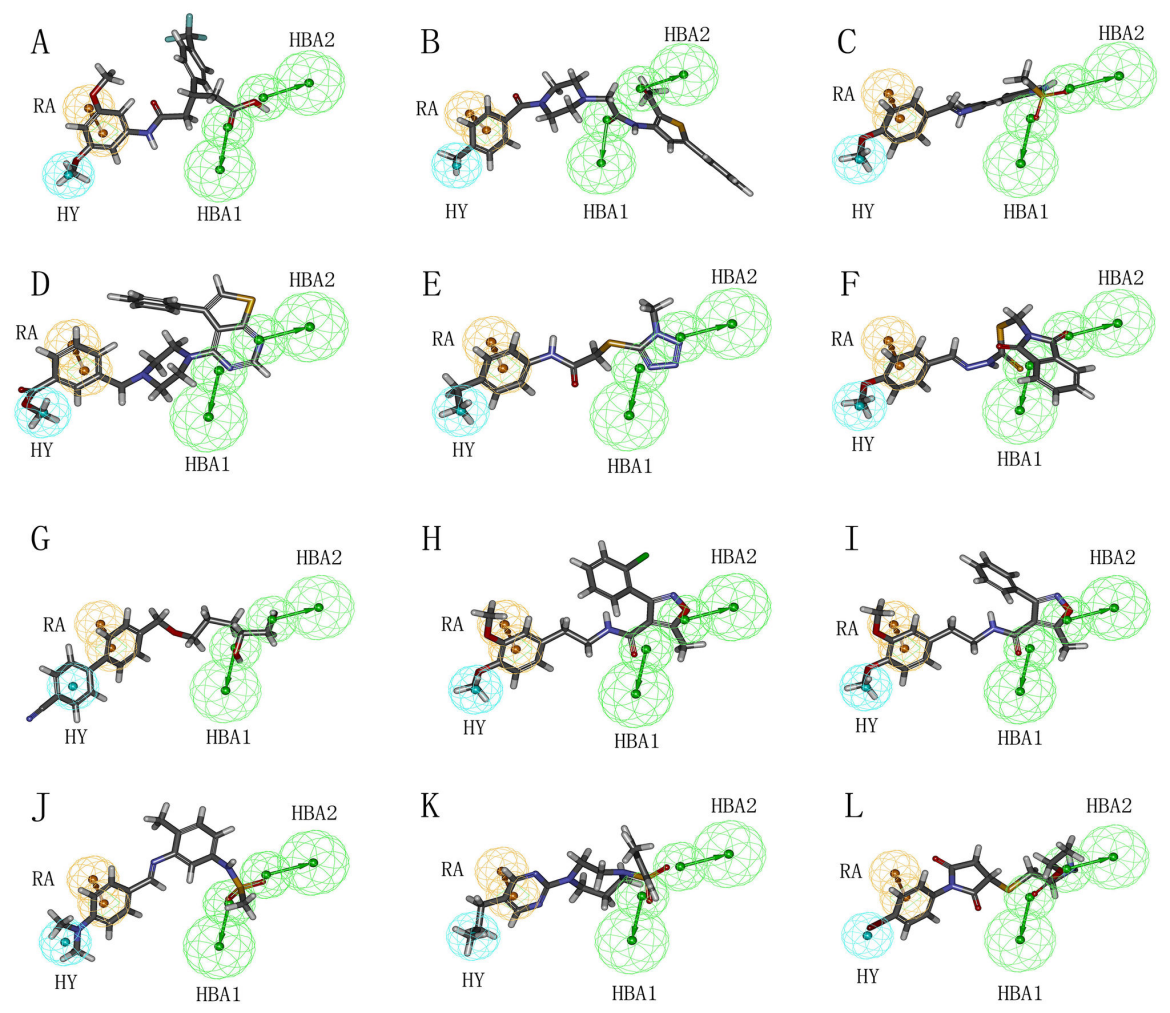
Pharmacophore mapping of twelve hit compounds based on the best pharmacophore model, Hypo1. (A) PD00519. (B) GK03776. (C) BTB01176. (D) AW01131. (E) AA-768-32245030. (F) AA-504-32628026. (G) AA-516-12432156. (H) BTB01888. (I) BTB01889. (J) BTB01180. (K) AW00861. (L) AA-768-30891048. Pharmacophore features are color-coded: cyan, hydrophobic (HY); orange, ring aromatic (RA); green, hydrogen bond acceptor (HBA).

**Figure 11:**
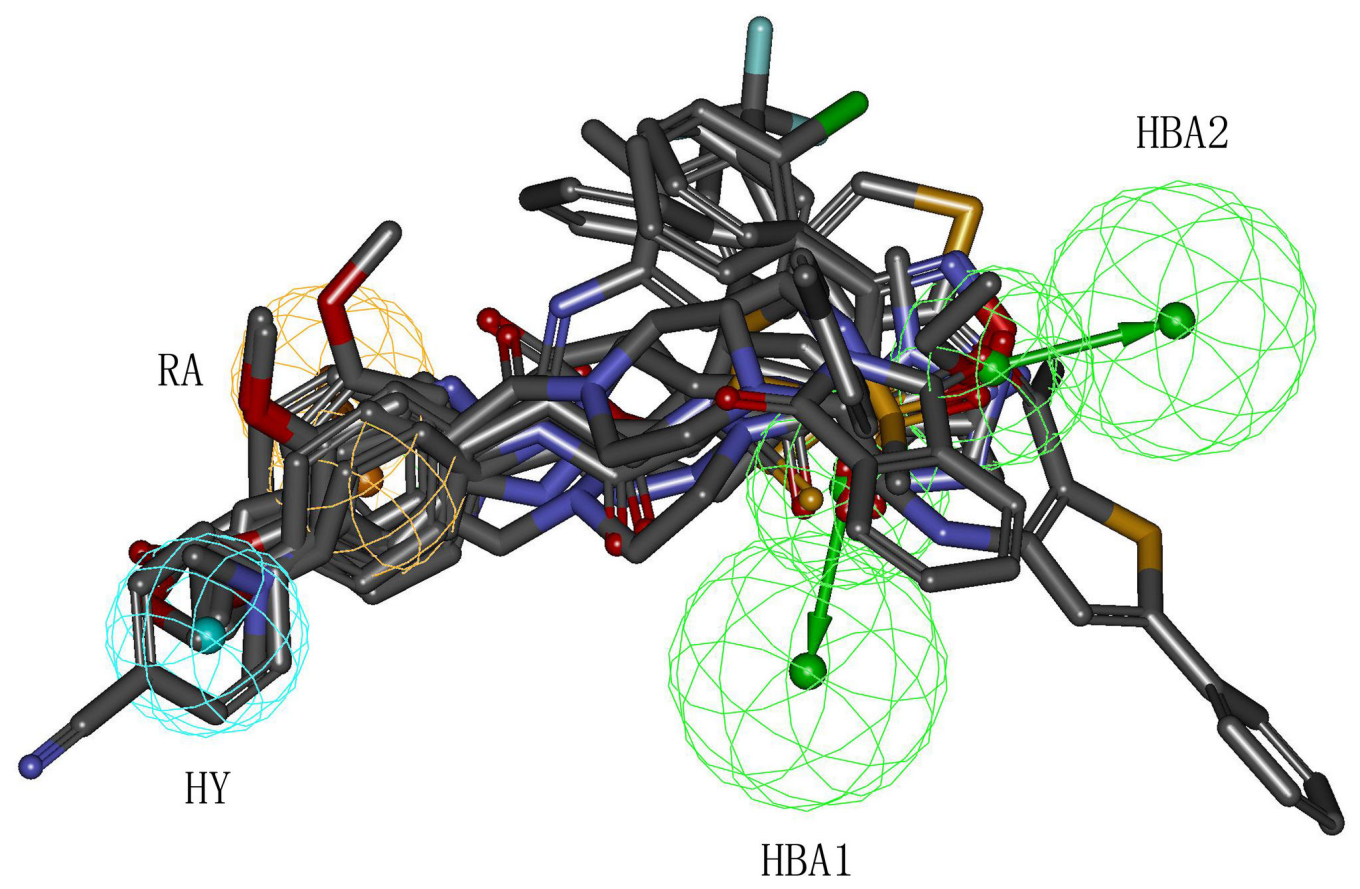
Alignment of twelve hit compounds with the best pharmacophore model, Hypo1. Pharmacophore features are color-coded: cyan, hydrophobic (HY); orange, ring aromatic (RA); green, hydrogen bond acceptor (HBA).

 The pharmacophore mapping of twelve hits on Hypo1 is depicted in Figure 10. Every hit compound has mapped four pharmacophoric features of Hypo1. Figure 11 shows the superimposition of twelve hit compounds on the Hypo1. The results indicated that hit compounds can produce perfect mapping with Hypo1. The benzene rings and oxygen atoms of hit compounds that overlaid the RA and HBA features of Hypo1, respectively, enabled considerable hydrophobic and polar interactions with the important amino acids in the active site. In addition, hydrophobic groups of these compounds that mapped the HY feature of Hypo1 interacted hydrophobically with the amino acids. Thus, in the design of potent inhibitors of PDE4, all twelve hit compounds which showed good results with respect to following properties, such as estimated activity, calculated drug-like properties and scores can be proposed as potential leads. Novelty search for compounds using SciFinder Scholar and PubChem search had also ascertained that these hits were not reported earlier for PDE4 inhibition. Therefore, we suggest that the identified compounds are novel and potent virtual leads for PDE4 inhibitor design. 

## Materials and Methods

### Pharmacophore model generation

HypoGen module of Discovery Studio program (DS), version 2.5, from Accelrys (San Diego, USA) was used to perform all pharmacophore modeling calculations. While 13 compounds were selected from one article [3], other fourty-three structurally diverse compounds with inhibitory activity (IC_50_) data were chosen as the training and test set compounds from similar articles [4-8] reported by same group of researchers. All the 56 compounds were tested for inhibition activity against PDE4 prepared from U937 cells (a cell line derived from human monocytes) by using the same experimental conditions. Based on the diversity of chemical structures and experimental activity values, we selected 28 compounds with wide activity range (0.051 to 4000 nM) as the training set. The two-dimensional (2D) chemical structures of all the compounds were built and subsequently converted to 3D structures in Discovery Studio program 2.5 (DS). For the compounds in the the data set, CHARMM forcefield was used to perform energy minimization process. Poling algorithm was used to generate a maximum of 230 diverse conformations with the energy threshold of 15 kcal mol^-1^ above the calculated energy minimum for every compound in the dataset [9,10]. Diverse Conformer Generation protocol running with Best/Flexible conformer generation option was applied to generate multiple conformers. By performing a more rigorous energy minimization in both torsional and cartesian space, this method ensures the best coverage of conformational space [11]. All the 28 compounds of training set were submitted to the HypoGen module of DS. The minimum and maximum count for all the features in the hypothesis run were set of 1 and 6, respectively. Uncertainty value was set to 2 and the minimum inter-feature distance was set to 2.5 Å from the default value of 2.97 Å. Hydrogen bond acceptor (HBA), hydrogen bond donor (HBD), hydrophobic (HY) and ring aromatic (RA) features were used to generate ten pharmacophore models using 3D QSAR pharmacophore generation of DS [9,10]. All other parameters used in HypoGen module were kept at their default settings [9,10]. In this study, the top 10 hypotheses returned by the hypotheses generation process were selected for further calculations.

### Pharmacophore model evaluation

Based on cost functions and other statistical parameters which were calculated by HypoRefine module during hypothesis generation, the quality of the generated pharmacophore models was evaluated. The best pharmacophore model should have a high correlation coefficient, low RMSD values and total cost that should be away from the null cost and close to the fixed cost [9,10]. All of these cost values are reported, and a difference of 40-60 bits between the total and null costs suggests a 75-90% chance of representing a true correlation in the data [9,10]. To investigate the ability to estimate the activity of new compounds, the selected pharmacophore model was further validated by three methods including test set method, Fischer’s randomization test and leave-one-out method. 28 diverse compounds were used as the test set to validate the pharmacophore model. For Fischer’s randomization test, 95% confidence level was chosen in this validation study and the 19 random spreadsheets were constructed. Finally, we performed the leave-one-out methodology for the cross validation of the model by using the same parameters as used for generating original pharmacophore model, thus 28 pharmacophore models were generated. To ensure the influence of each compound from the training set in the generation of selected pharmacophore model, one compound at a time from 28 compounds was left [10-13].

### Virtual screening

In order to identify novel hit compounds, the best pharmacophore model after validation was used as 3D structural search query to screen three chemical databases including Specs (135556 molecules), Maybridge (59652 molecules) and NCI (238819 molecules), respectively. Search 3D Database protocol with Best/Flexible search option was applied in database screening. The hits identified through database screening were further filtered using estimated activity, Lipinski’s rule of five [14], and ADMET properties [15-18]. A Lipinski-positive compound has (i) a molecular weight < 500; (ii) < 5 hydrogen bond donor groups; (iii) < 10 hydrogen bond acceptor groups and (iv) an octanol/water partition coefficient (Log P) value < 5 [9,10].

### Molecular docking

The docking study was performed using the Molecular Operating Environment (MOE) software (Chemical Computing Group Inc.). The crystal structure of PDE4 obtained at a resolution of 1.72 Å was downloaded from the protein data bank (PDB entry: 1XON). This structure was then protonated in the Molecular Operating Environment (MOE) via MMFF94x force field. The hits identified through database screening were subjected to molecular docking studies. The active site was defined with a 6 Å radius around the bound inhibitor. The triangle matcher algorithm of the MOE software packages was used to dock the identified hits into the protein active site. According to this algorithm, different poses were generated by aligning ligand triplets of atoms on triplets of alpha spheres. For all scoring functions, lower scores indicated more favorable poses. The scoring function of these compounds has to obey the following parameters: (1) Specifying ASE Scoring to use for ranking the poses output by the placement stage; (2) Specifying Forcefield Refinement to use to relax the poses, respectively; (3) Specifying Affinity dG Scoring to use for ranking the poses output by the refinement stage. The free energy of binding was calculated from the contributions of hydrogen bond, ionic, hydrophobic and van der Waals interactions between the protein and ligand, intramolecular hydrogen bonds and strains of the ligand. We observed in the S field that the docking poses were ranked by the binding free energy calculation.

## Conclusions

In the present work, a highly correlating (r = 0.963930) pharmacophore model (Hypo1) containing two hydrogen bond acceptors, one hydrophobic region and one aromatic ring feature, was selected through various parameters such as total cost, correlation coefficient and cost difference. Further validation was done by using test set prediction, Fischer randomization method and leave-one-out method. Result of these validation tests showed that Hypo1 could accurately predict the active compounds, it has better statistical values compared to other randomly generated pharmacophore models and its correlation coefficient is not solely depended on a single compound. This validated Hypo1 was led to database screening for identifying compounds which can be used as potent PDE4 inhibitor design. Further studying these compounds by drug-like filtrations and molecular docking, also suggested the robustness of Hypo1. In the end, twelve structurally diverse compounds having high estimated activity and strong molecular interactions with key active site amino acids of PDE4 were identified. Therefore, the results of this study will assist, not only in the development of new potent hits for PDE4, but also in providing a better understanding of the interaction between PDE4 and inhibitors. This will in turn be beneficial to the rational design of novel potent enzyme inhibitors.

## Supporting Information

Text S1 **Pharmacophore modelling and 3D database search.**
(DOC)

Text S2 **The steps of the procedure in our study.**
(DOC)

Figure S1 **The Chemical structures of twelve hit compounds from databases.**
(TIF)
