# Supplementary material for: Pharmacophore Modeling and Virtual Screening for the Discovery of New type 4 cAMP Phosphodiesterase (PDE4) Inhibitors
Source: PLoS One. 2013 Dec 10;8(12):e82360. doi: 10.1371/journal.pone.0082360 (PMC3858292; doi:10.1371/journal.pone.0082360)
Supplement: Text S1 — Pharmacophore modelling and 3D database search. (DOC) [file pone.0082360.s001.doc]

**Text S1 Pharmacophore modelling and 3D database search.**

To identify the novel and diverse leads, a highly correlating (r = 0.963930) pharmacophore model after successful validation is used in database screening to identify chemical compounds. Screened compounds have been subjected to various drug-like filtrations and molecular docking studies. Finally, twelve potential inhibitory leads with better scores and interactions with key active site amino acids are identified to be used in designing novel PDE4 inhibitors as inflammatory drugs.
