# Supplementary material for: Pharmacophore Modeling and Virtual Screening for the Discovery of New type 4 cAMP Phosphodiesterase (PDE4) Inhibitors
Source: PLoS One. 2013 Dec 10;8(12):e82360. doi: 10.1371/journal.pone.0082360 (PMC3858292; doi:10.1371/journal.pone.0082360)
Supplement: Text S2 — The steps of the procedure in our study. (DOC) [file pone.0082360.s002.doc]

**Text S2 The steps of the procedure in our study.**

(1) Developed first three-dimensional pharmacophore (3D) model for PDE4 inhibitors. (2) Model used in database screening to identify novel hit compounds. (3) Hit compounds subjected to drug-like filtrations and molecular docking studies. (4) Twelve leads identified to be used in designing PDE4 inhibitors as inflammatory drugs.
