# Supplementary figures and images for: Pharmacophore Modeling and Virtual Screening for the Discovery of New type 4 cAMP Phosphodiesterase (PDE4) Inhibitors
Source: PLoS One. 2013 Dec 10;8(12):e82360. doi: 10.1371/journal.pone.0082360 (PMC3858292; doi:10.1371/journal.pone.0082360)

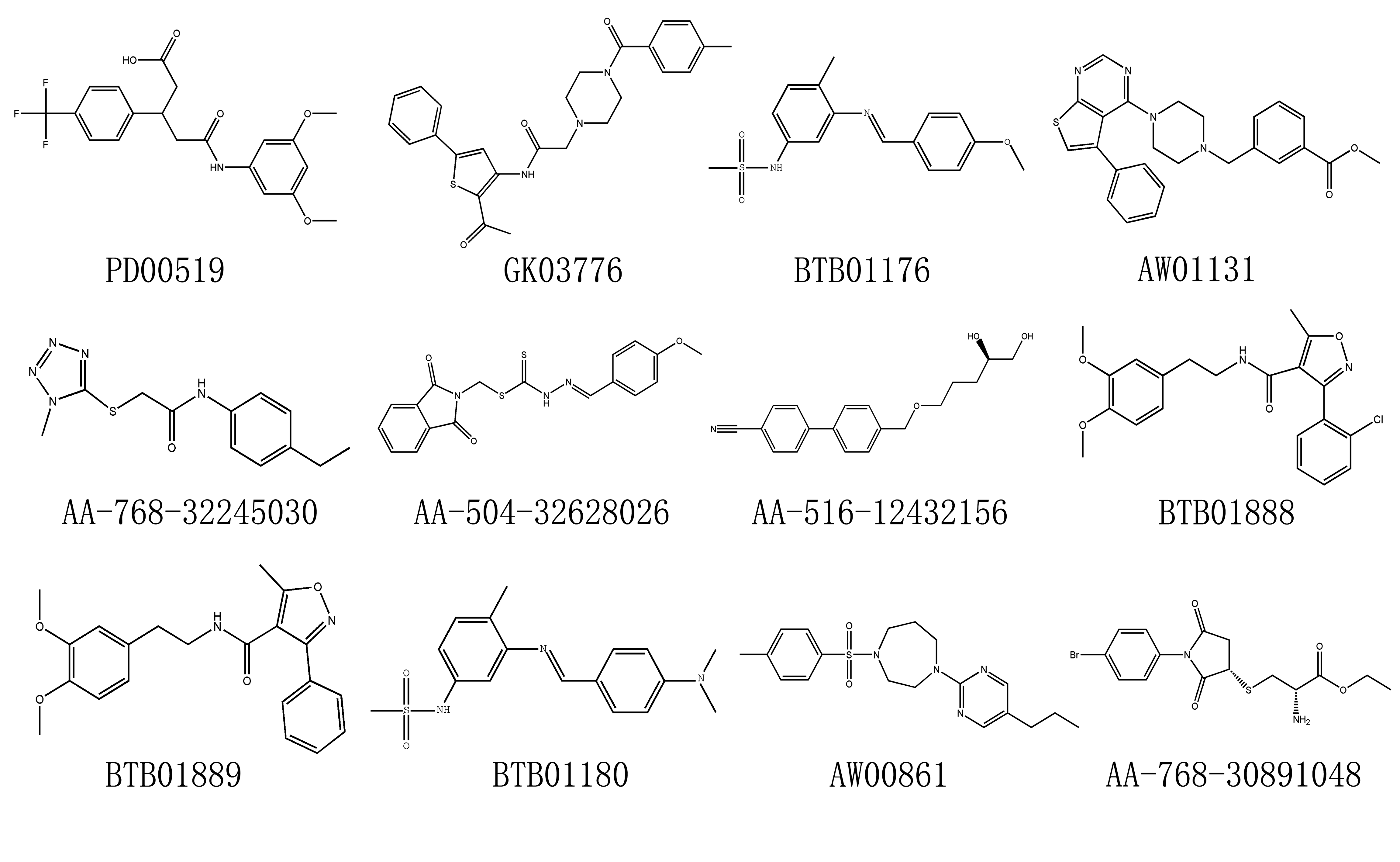

Supplement: Figure S1 — The Chemical structures of twelve hit compounds from databases. (TIF) [file pone.0082360.s003.tif]
